# Greater tolerance of uncertainty facilitates thriving in doctors entering postgraduate training

**DOI:** 10.1186/s12909-025-07645-2

**Published:** 2025-07-16

**Authors:** Russell Peek, Rachel Arnold, Lee Moore

**Affiliations:** 1 Department for Health, University of Bath, Bath, UK; 2 Gloucestershire Hospitals NHS Foundation Trust, Gloucester, UK

**Keywords:** Human thriving, Medical education, Strain, Tolerance of Uncertainty, Transition

## Abstract

**Background:**

Medical curricula increasingly emphasise the need to prepare graduates to manage uncertainty. Uncertainty is an inevitable consequence of the complex nature of human health and illness but may be stressful for clinicians less able to tolerate it. Although work-related stress is prevalent in healthcare services, not all clinicians struggle under the pressures they face. Indeed, some thrive, experiencing success and development, observed through the joint experience of high levels of wellbeing and perceived performance. Therefore, this study aimed to explore relationships between tolerance of uncertainty, perceived stress, and performance and wellbeing (i.e., human thriving) in doctors entering UK foundation training.

**Methods:**

Sixty-six doctors entering UK foundation training completed validated self-report measures to assess tolerance of uncertainty, perceived stress, wellbeing and performance. Multiple linear regression analyses were used to investigate relationships between tolerance of uncertainty, perceived stress, and human thriving. Mediation analysis was then undertaken to explore whether the observed relationship between tolerance of uncertainty and thriving was mediated by perceived stress.

**Results:**

Tolerance of uncertainty and perceived stress predicted a significant proportion of variance in thriving, independently of sex, lifetime stress, and adverse childhood experiences (model adjusted *R*^2^ = 0.51). Additionally, tolerance of uncertainty accounted for a significant proportion of variance in perceived stress after controlling for covariates (model adjusted *R*^2^ = 0.43). Furthermore, the effect of tolerance of uncertainty on thriving was partially mediated by perceived stress.

**Conclusions:**

Tolerance of uncertainty may represent a novel enabler of thriving and act, at least in part, by reducing perceived stress. Medical educators should consider how best to equip doctors in training to face uncertainty in clinical practice. Further research is required to examine whether interventions can optimise tolerance of uncertainty, or reduce perceived stress in uncertain situations, to facilitate thriving in early career doctors.

**Supplementary Information:**

The online version contains supplementary material available at 10.1186/s12909-025-07645-2.

## Background

Human thriving is “the joint experience of development and success, which can be realised through effective holistic functioning and observed through the experience of a high-level of wellbeing and a perceived high-level of performance” [1, p.175]]. This definition draws upon and integrates conceptualisations of thriving across disciplinary contexts and over time [1, 2]. For example, in occupational psychology, thriving is characterised by a joint sense of learning and vitality at work [3]. In developmental domains (e.g., positive youth development) researchers consider thriving a growth-oriented process, while in performance domains (e.g., business), thriving has been conceptualised as a sense of accomplishment, prosperity, success, and wealth (see, for a review, [1]). While bringing together disciplinary perspectives, Brown and colleagues [1] draw an important distinction between thriving and terms that may appear similar, such as prospering, resilience, and flourishing. While aspects of these constructs overlap with thriving, they do not capture the combined experience of both development and success without the need for preceding adversity. Previous studies have identified enablers that may facilitate thriving: both contextual (e.g., supportive co-workers, effective leadership) and personal (e.g., proactive personality, work engagement) [2, 3]. However, empirical evidence for enablers and inhibitors of thriving remains limited, with an identified need for further research [3].

Workforce wellbeing and performance (i.e. thriving) is central to the delivery of effective healthcare services [4]. However, in the United Kingdom (UK), the annual National Health Service (NHS) staff survey paints a concerning picture of workforce wellbeing. In 2023, 41.7% of NHS staff reported feeling unwell as a result of work-related stress, with an even higher rate amongst doctors in training (48.9%) [5]. In its annual report on the state of UK medical education and practice, the General Medical Council (GMC) highlighted increasing clinical pressures exacerbating chronic challenges to doctors’ mental health and welfare, with potential impacts on patient safety and workforce retention [6]. The problem is not unique to the UK, with international studies highlighting the high prevalence of work-related stress in healthcare professionals (e.g., from 18% of General Practitioners in the Netherlands to 56% in Sweden) [7, 8]. Furthermore, recent reports raise concern about declining productivity despite increased funding and staffing [9]. Despite such evidence of suboptimal wellbeing and performance across the workforce, it is notable that not all clinicians struggle under the pressures they face. Indeed, some thrive [10]. This raises important questions about the cause and nature of these pressures, and what allows some clinicians to thrive despite them.

Undergraduate and postgraduate medical curricula increasingly emphasise the need to prepare graduates to manage uncertainty, recognising the ubiquity and impact of uncertainty in clinical practice (e.g.,). Uncertainty is sometimes viewed simply as a result of insufficient technical, conceptual, or personal knowledge, making it definable and potentially remediable [11–13]. However, uncertainty can also stem from metaphysical complexity (what is possible to know), probability effects (random variation), or moral ambiguity (what is ‘right’), none of which can be easily resolved with more information. Inherent ambiguity, complexity, and random variation in human health and illness make uncertainty inevitable, while the culture of medicine demands certainty [14]. This misalignment between cultural expectations and personal experience can have a psychological impact, leading to fear, worry, perceptions of vulnerability, and avoidance of decision-making [15]. Uncertainty contributes to many known challenges to the wellbeing and performance of doctors in training, including prioritisation, effective teamworking, decision-making, managing self-directed learning, and dealing with work-related pressures [16–18]. These challenges are particularly acute during transition between stages of training, which requires adaptation to new relationships, routines, assumptions, roles, and expectations [18, 19].

Tolerance of uncertainty is an individual’s capacity to endure an aversive response triggered by the perceived absence of salient, key, or sufficient information, and the associated perception of uncertainty [20]. This definition implies that individuals experience an acute stress response in situations where uncertainty exceeds a threshold of endurance (i.e., a stress response is activated where uncertainty becomes intolerable). However, the Generalised Unsafety Theory of Stress (GUTS) holds that the experience and psychophysiological effects of stress are the result of failure to inhibit a default stress response (i.e., failure to turn ‘off’), rather than a response to activation by a trigger (i.e., turned ‘on’) [21]. Evidence from neurobiological research and evolutionary theory supports the notion that a default stress state is inhibited by a largely subconscious perception of safety [22]. From this perspective, individuals will experience stress in situations of uncertainty unless they feel safe despite the uncertainty. This association between uncertainty and a perceived lack of safety is consistent with evidence from studies of people experiencing anxiety disorders, in whom *in*tolerance of uncertainty is common, and for whom anxiety is experienced in the presence of uncertainty and a perceived threat [23].

A review of 11 studies found that lower tolerance of uncertainty (or ambiguity) was associated with poorer wellbeing in medical training [24]. However, ‘wellbeing’ in the included studies was assessed using measures of burnout, emotional exhaustion, depression, perceived stress, or psychological distress. This is problematic, as wellbeing is not merely the absence of mental ill-health, but a state of positive feelings and meeting full potential [25]. Indeed, the World Health Organisation (WHO) identifies wellbeing as encompassing “…quality of life, as well as the ability of people and societies to contribute to the world in accordance with a sense of meaning and purpose” (24, p10). No studies included in Hancock and Mattick’s review [22] directly assessed wellbeing as a positive psychological state (e.g., via indices of positive affect, life satisfaction, vitality). There remains, therefore, a gap in our understanding of the relationship between tolerance of uncertainty and wellbeing in medical learners.

Furthermore, individuals with low tolerance of uncertainty may function less well in uncertain situations, with impairment of performance. Thibodeau et al. [27] observed a negative relationship between *in*tolerance of uncertainty and typing speed, and Carleton et al. [28] reported a positive relationship between *in*tolerance of uncertainty and risk-averse decision-making in behavioural tasks (e.g., Wisconsin Card Sorting Task). Despite evidence that tolerance of uncertainty affects performance in controlled tasks, there has been little research to date into the impact of tolerance of uncertainty on perceived or objectively assessed performance in real-world situations (e.g., medical training). Performance of doctors in training may be affected by strategies adopted to cope with uncertainty, which may be adaptive (e.g., seeking help) or maladaptive (e.g., becoming task focused to avoid complex decision-making [29]. Existing research suggests that individuals who are *in*tolerant of uncertainty are more likely to demonstrate biased information processing in ambiguous situations and rely more on affective inputs for making decisions, such as the perceived attractiveness of an option rather than its functionality [30, 31]. Relationships between uncertainty and emotion are complex, however, as uncertainty can enhance the intensity of negative emotions (e.g., anxiety) or positive emotions (e.g., excitement), depending on the valence of an anticipated outcome (i.e., potential gain or loss; [32]).

Our understanding of the relationships between tolerance of uncertainty, perceived stress, and performance and wellbeing (i.e., human thriving) is thus restricted by a relatively limited evidence base. To extend existing research, this study investigated these relationships in doctors making the transition from medical school to postgraduate training in the UK foundation programme, a two-year work-based training programme intended to bridge the gap between medical school and specialty or general practice training.

As previous studies suggest this transition exposes doctors to significant uncertainty, it was hypothesised that:

1. Since both wellbeing and performance may be affected by tolerance of uncertainty [24, 27], greater tolerance of uncertainty would be positively associated with thriving (i.e., high levels of wellbeing and perceived high levels of performance; [1];
2. Consistent with our theoretical framework (i.e., GUTS; [21]), tolerance of uncertainty would be inversely related to perceived stress, and;
3. Given that contextual and personal factors can enable or inhibit thriving [3], that tolerance of uncertainty shows interpersonal variation [15], and that uncertainty is inevitable in clinical practice and may be stressful [14, 33], the relationship between tolerance of uncertainty and thriving would be mediated by perceived stress, such that greater tolerance of uncertainty would facilitate thriving via lower perceived stress.

## Methods

### Participants

Based on the results of previous studies examining tolerance of uncertainty, perceived stress, and mental ill-health [34–36], a positive correlation of approximately *r* =.30 was anticipated between tolerance of uncertainty and thriving. For bivariate linear regression of tolerance of uncertainty on thriving, an a priori power calculation in G*Power software [37] suggested at least 64 participants would be required, given an alpha of 0.05 and power of 80%. In keeping with this sample size estimate, a sample of 66 doctors in training (42 female, 22 male, and 2 who preferred not to say) took part, with a mean age of 25.4 (*SD* = 2.0) years. Participants had graduated from 23 different medical schools in the UK and internationally and were employed in a range of acute and community NHS healthcare services.

### Study design and procedure

Following institutional approval, doctors entering a UK regional foundation training programme in August 2022 and 2023 were invited to participate by email. Informed consent was obtained after participants reviewed information about the study and had the opportunity to ask questions. A secure, web-based questionnaire (supplemental information, presented via www.onlinesurveys.ac.uk) was used to present valid and reliable measures, described below, to assess tolerance of uncertainty, perceived stress, and perceived performance and wellbeing (subsequently combined into a measure of human thriving). The questionnaire took ~ 20 min to complete.

### Measures

#### Tolerance of uncertainty

Tolerance of uncertainty was assessed using the Tolerance of Ambiguity in Medical Students and Doctors (TAMSAD) scale [38], which includes 29 clinically contextualised items (e.g., “I feel comfortable that in medicine there is often no right or wrong answer”). The terms ambiguity and uncertainty are often used interchangeably in the literature, but ambiguity, complexity, and probability are better viewed as properties of a stimulus that make it difficult to completely understand, and uncertainty as an individual’s awareness of incomplete information on which to act [15, 39]. Although the TAMSAD scale title suggests measurement of tolerance of ambiguity rather than uncertainty, items reflect the experience of uncertainty rather than ambiguity as a precipitating cause (e.g., “I feel apprehensive when faced with a new clinical situation or problem”). Agreement with each item was scored on a 5-point Likert scale ranging from 1 (*strongly disagree*) to 5 (*strongly agree*). Consistent with past research [38], negatively-worded items were reverse scored before a 0-100 score was calculated by linear transformation of the mean item score using the formula: 25*(M_item_-1). A higher score reflected greater tolerance of uncertainty. The scale demonstrated satisfactory reliability and validity in a previous study [38], and acceptable internal consistency in the current study (Cronbach’s α = 0.83) [40].

#### Perceived performance

Perceived performance was assessed using the Subjective Career Success Inventory (SCSI) [41]. This inventory consists of 24 items (e.g., “I am proud of the quality of the work I have produced”, “The organisations I worked for have recognised me as a good performer”) and measures personal career success across eight dimensions (i.e., recognition, quality work, meaningful work, influence, authenticity, personal life, growth and development, and satisfaction). Each item was scored on a 5-point Likert scale ranging from 1 (*strongly disagree*) to 5 (*strongly agree*). All item scores were summed (range = 24 to 120), with a higher score reflecting greater perceived performance. The SCSI has demonstrated validity and reliability [42], and demonstrated acceptable internal consistency in the current study (Cronbach’s α = 0.90) [40].

#### Wellbeing

Wellbeing was assessed via the Warwick-Edinburgh Mental Well-Being Scale (WEMWBS) [43]. The scale includes 14 items exploring hedonic and eudaimonic aspects of wellbeing, including positive affect (e.g., “I’ve been feeling good about myself”), satisfying interpersonal relationships (e.g., “I’ve been feeling close to other people”), and positive functioning (e.g., “I’ve been dealing with problems well”). For each item, personal experience over the past two weeks was rated on a 5-point Likert scale ranging from 1 (*none of the time*) to 5 (*all the time*). All item scores were summed (range = 14 to 70), with a higher score indicating greater well-being. The WEMBWS demonstrated acceptable internal consistency in the current study (Cronbach’s α = 0.94) [40].

#### Human thriving

A composite human thriving score was calculated as the sum of standardised values (i.e., z-scores) for perceived performance (i.e., SCSI) and wellbeing (i.e., WEMWBS), with each given equal weighting. A similar approach has been used previously in a sporting context, utilising context-relevant measures of performance and wellbeing [44]. The use of a composite score reflects the theoretical construct of human thriving as the joint experience of development and success, determined through high levels of perceived performance and wellbeing.

#### Perceived stress

The 10-item version of the Perceived Stress Scale (PSS) [45] was used to explore how frequently participants experienced 10 thoughts or feelings over the last month (e.g., “How often have you felt you were unable to control the important things in your life”, “How often have you felt that you were on top of things”). Each item was scored on a 5-point Likert scale ranging from 0 (*never*) to 4 (*very often*). Positively-worded items were reverse scored before all items were summed (range = 0 to 40), with a higher score reflecting greater perceived stress. The scale has been shown to be valid and reliable [46], and demonstrated acceptable internal consistency in the current study (Cronbach’s α = 0.89) [40].

#### Confounding factors

Lifetime stress, sex, and Adverse Childhood Experiences (ACEs) were considered potential covariates that may explain some inter-individual variation in perceived stress or human thriving. Participants were asked to self-report lifetime experience of stress on a 7-point Likert scale ranging from 0 (*none*) to 6 (*extreme amount*), in response to the prompt “How much stress have you experienced throughout your life?”. A similar single-item holistic assessment of stress has been used in past research [47]. Wellbeing and perceived stress have shown sex differences in previous research [43, 48], while TAMSAD scores have not [38]. ACEs are strongly related to future health and wellbeing [49]. Two yes/no items were also used to identify participants who had experienced childhood adversity. Specifically, participants were asked “When growing up, did you live with anyone who was a problem drinker or alcoholic?” and “Did parents or adults in your home swear at you, insult you, or put you down more than once?”. Compared to the full 11-item Behavioural Risk Factor Surveillance System ACEs measure [50], a positive answer (i.e., yes) to both these questions has a sensitivity of 70% and specificity of 94% for identifying significant childhood adversity. Further, this two-item tool has similar associations with a range of health outcomes as the full 11-item measure (e.g., obesity, smoking; [50]).

### Data analysis

Data were analysed in SPSS v28 (IBM, 2021). The dataset was screened for missing and outlying values. There were no missing data for tolerance of uncertainty, wellbeing, or perceived performance. However, perceived stress data were missing for six participants. In this instance, pairwise deletion was used in subsequent analyses. The distribution of each variable was assessed visually using histograms and QQ plots, and statistically with significant departure from normality indicated by absolute skew > 2 or kurtosis > 7 [51]. Potential univariate outliers were identified using a z-score cut-off of ± 3. Applying these checks, a single outlier was identified for tolerance of uncertainty and then winsorized to a value 3 standard deviations above the mean. All variables were subsequently normally distributed.

Next, descriptive statistics and intercorrelations were calculated. Bivariate linear regression analyses were then conducted to examine if variance in perceived stress or thriving could be attributed to tolerance of uncertainty, and if variance in thriving could be accounted for by perceived stress. Forced entry multiple linear regression analysis was then conducted to determine whether relationships between tolerance of uncertainty, perceived stress, and thriving were robust to controlling for sex, lifetime stress, and ACEs as potential confounding factors [52]. Residual plots were examined to confirm validity of each regression model and alpha level was set at *p* <.05. Finally, mediation analysis was undertaken to examine if the effect of tolerance of uncertainty on thriving was mediated by perceived stress, using model four of the PROCESS dialog for SPSS [53] and 5000 bootstrap samples to determine 95% confidence intervals.

## Results

Descriptive statistics and intercorrelations for all variables are presented in Table 1.

**Table 1:** Descriptive statistics and intercorrelations for all variables

Variable (scale range)	Mean	SD	Range	Perceived stress	Tolerance of uncertainty	Perceived performance	Mental wellbeing
Perceived stress (0–40)	17.22	6.66	28.00				
Tolerance of uncertainty (0-100)	58.26	9.00	47.20	− 0.40^**^			
Perceived performance (24–120)	79.02	12.04	66.00	− 0.47^**^	0.33^**^		
Mental wellbeing (14–70)	47.80	9.31	39.00	− 0.81^**^	0.44^**^	0.61^**^	
Human thriving	0.00	1.80	9.13	− 0.71^**^	0.43^**^	0.90^**^	0.90^*^

*Notes*: * and ** correlations significant at 0.05 and 0.01 level, respectively (two-tailed)

Results of bivariate linear regression analyses are shown in Table 2. In relation to Hypothesis 1, the regression model revealed a significant relationship between tolerance of uncertainty and human thriving (F [1, 64] = 14.29, *p* <.001), with an R^2^ of 0.18, suggesting that tolerance of uncertainty explained 18% of the variance in thriving. A similarly significant relationship was found between perceived stress and thriving, (F [1, 58] = 60.37, *p* <.001), with R^2^ of 0.51, indicating 51% of the variance in thriving could be explained by perceived stress. Finally, in relation to Hypothesis 2, the relationship between tolerance of uncertainty and perceived stress was statistically significant (F [1, 58] = 10.95, *p* =.002), with an R^2^ of 0.16, implying that tolerance of uncertainty explained 16% of the variance in perceived stress.

**Table 2:** Results of bivariate linear regression analyses

Dependent variable	Independent variable	B [95% CI]	SE B	β
Human thriving	Tolerance of uncertainty	0.09 [0.04, 0.13]	0.02	0.43^**^
	Perceived stress	− 0.19 [-0.24, -0.14]	0.02	− 0.71^**^
Perceived stress	Tolerance of uncertainty	− 0.29 [-0.47 to -0.12]	0.09	− 0.40^**^

*Notes*: CI = Confidence Interval. * *p* <.05, ** *p* <.01, *** *p* <.001

In forced entry multiple linear regression analysis (Table 3), relationships between tolerance of uncertainty (*β* = 0.22, *p* =.038), perceived stress (*β* = − 0.59, *p* <.001), and thriving remained significant after accounting for sex, lifetime stress, and ACEs as potential confounding factors (F [5, 53] = 12.85, *p* <.001, adjusted *R*^2^ = 0.51). The relationship between tolerance of uncertainty and perceived stress also remained significant (*β* = 0.25, *p* =.023). As well as tolerance of uncertainty, the regression model demonstrated significant effects of sex, lifetime stress, and ACEs on perceived stress (F [4, 53] = 11.44, *p* <.001, adjusted *R*^2^ = 0.43).

**Table 3:** Results of forced entry multiple linear regression analyses

Dependent variable	Independent variables	B	SE B	β
		[95% CI]		
Human thriving	Tolerance of uncertainty	0.04 [0.00 to 0.08]	0.02	0.22^*^
	Perceived stress	-0.16 [-0.22 to -0.09]	0.03	− 0.59^***^
	Sex	-0.02 [-0.65 to 0.62]	0.31	− 0.01
	Lifetime stress	-0.06 [-0.33 to 0.20]	0.13	− 0.05
	Adverse childhood experiences	-0.26 [-1.65 to 1.14]	0.70	− 0.04
Perceived Stress	Tolerance of uncertainty	-0.18 [-0.33 to -0.03]	0.08	− 0.25^*^
	Sex (male)	-3.05 [-5.97 to -0.13]	1.46	− 0.22^*^
	Lifetime stress	1.98 [0.97 to 3.00]	0.51	0.28^*^
	Adverse childhood experiences	7.44 [2.07 to 12.81]	2.68	0.41^***^

*Notes*: CI = Confidence Interval. * *p* <.05, ** *p* <.01, *** *p* <.001

**Fig. 1:**
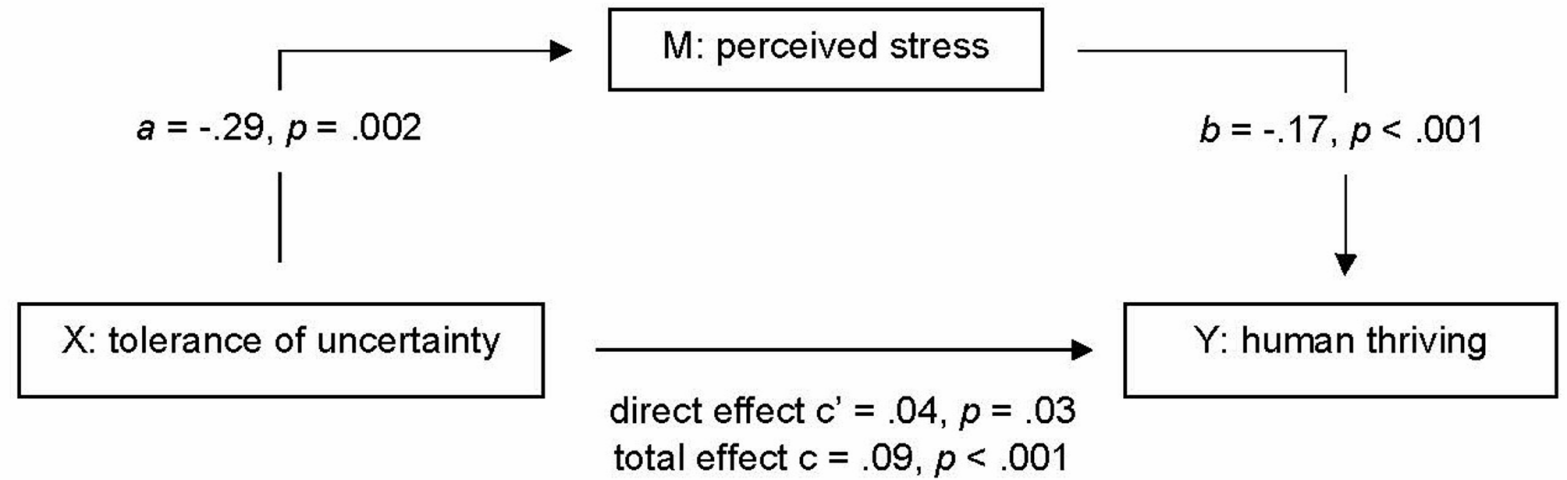
Mediation path diagram

Given the significant effect of tolerance of uncertainty on perceived stress, and of perceived stress on thriving, mediation analysis was performed to assess if the effect of tolerance of uncertainty on thriving was mediated by perceived stress (Hypothesis 3). Results suggested that perceived stress partially mediated the relationship between tolerance of uncertainty and thriving (standardised indirect effect, *β* = 0.25, 95% CI = 0.11 to 0.41) (Fig. 1).

## Discussion

To our knowledge, this is the first study to investigate relationships between tolerance of uncertainty, perceived stress, and human thriving. In a cohort of doctors making the transition from medical school to postgraduate training, relationships were found between tolerance of uncertainty and perceived stress and thriving, even after controlling for sex, lifetime stress, and ACEs. Thus, tolerance of uncertainty may represent a novel enabler of thriving. Furthermore, mediation analysis showed that the relationship between tolerance of uncertainty and thriving was partially mediated by perceived stress (i.e. greater tolerance of uncertainty can enable thriving partly by lowering perceived stress). Given the positive effects of thriving on both the individual and organisation in which they work [54, 55], and the high rates of work-related stress in healthcare workers [5], these results have important implications for clinicians, healthcare organisations, and future research.

We tested the hypothesis that tolerance of uncertainty would be positively associated with, and thus an enabler of, human thriving (i.e., joint experience of development and success, observed through the experience of high levels of wellbeing and perceived performance; [1]). As hypothesised, the novel results demonstrated a significant positive relationship, such that greater tolerance of uncertainty enabled thriving. Previous studies have found that lower tolerance of uncertainty is associated with a higher incidence of poor psychological outcomes, such as anxiety, depression, and burnout [24]. The current study extends our understanding of the psychological effects of tolerance of uncertainty by demonstrating that greater tolerance is associated with experiencing greater wellbeing. This is an important distinction, since wellbeing is a positive psychological state and not merely the absence of psychological ill-health [26]. While many factors can influence the wellbeing of doctors in postgraduate training (e.g., access to social support networks, work intensity, rota design [51]), the results suggest that tolerance of uncertainty may be an important contributor to the variation in wellbeing reported by doctors in training, despite universal exposure to uncertainty [56, 57].

The observed positive relationship between tolerance of uncertainty and perceived performance suggests that an ability to cope with uncertain situations is important to self-perception of performance at work. This adds to evidence from controlled laboratory settings of a positive association between tolerance of uncertainty and objective performance in behavioural tasks requiring decision-making in conditions of uncertainty [27, 28]. Studies of the transition from medical student to doctor highlight a need to translate learning into action; moving from ‘what to do’ to ‘how to do it’. Yardley et al. [58] report that learning to manage uncertainty and fostering active decision-making in uncertainty aid the transition into clinical practice. It has been suggested that contextual and personal factors enable thriving through satisfaction of basic psychological needs, including autonomy (i.e., need to determine one’s own behaviour and actions), competence (i.e., need to achieve desired outcomes), and relatedness (i.e., need to feel connected to other people) [3, 59]. It is possible, therefore, that greater tolerance of uncertainty increases an individual’s confidence and self-perceived competence in uncertain situations [15]. Greater autonomy may be perceived if an individual is able to make decisions independently in uncertain situations, rather than adopt coping strategies that potentially reduce autonomy (e.g., seeking help or avoiding complex decision-making [29]). Thus, it is conceivable that tolerance of uncertainty enables thriving through satisfaction of basic psychological needs, although further research is required to test this hypothesis.

There is an established relationship between low tolerance of uncertainty and emotional disorders (e.g., generalised anxiety disorder) [60, 61]. Consistent with this relationship, past research has shown an association between low tolerance of uncertainty and psychological ill-health (e.g., depression) in doctors in training [24]. However, the results of the present study go further, revealing an inverse relationship between tolerance of uncertainty and perceived stress in individuals across a range of self-reported mental wellbeing (e.g., without ill-health). Consistent with our underlying theoretical framework (i.e., GUTS [21]), greater tolerance of uncertainty may permit individuals to perceive safety even when facing uncertain situations, thereby inhibiting a default stress response. The novel finding of partial mediation of the relationship between tolerance of uncertainty and human thriving by perceived stress suggests that tolerance of uncertainty does not enable thriving solely through satisfaction of basic psychological needs but, at least in part, through lowering perceived stress. The mechanism by which tolerance of uncertainty reduces perceived stress warrants further exploration.

Previous research has explored relationships between stress appraisal and thriving. Brown et al. [59] found that thriving is facilitated by a challenge appraisal (i.e., the perception that personal coping resources meet or exceed situational demands). While items within the PSS are not explicitly related to the context of uncertainty, they assess the frequency of experiencing emotional responses to everyday events, such as feeling angered by things beyond personal control, the perceived ability to overcome difficulties, and to cope with tasks and problems [45]. To explain our findings, it is possible that individuals with greater tolerance of uncertainty are more likely to appraise uncertain situations as challenging rather than threatening, perceiving that they can meet the demands placed upon them. In contrast, those who are less tolerant of uncertainty may appraise uncertain situations as threatening and unsafe, and aim to avoid them by seeking certainty or reassurance, potentially reinforcing avoidance behaviour and uncertainty intolerance [62–64]. Consistent with the observed relationships between tolerance of uncertainty, perceived stress, and thriving, challenge appraisals have been linked to more adaptive psychophysiological responses to stress (e.g., patterns of cardiac reactivity; [65]), superior performance under pressure [66], and better mental health and wellbeing [67]. It is conceivable, therefore, that tolerance of uncertainty enables thriving by increasing the likelihood of challenge appraisal in uncertain situations. This hypothesis requires testing in future research.

While it is clear that work-related stress and poor wellbeing are widespread concerns in healthcare, there is limited evidence for effective preventative or restorative interventions [68, 69]. Strategies tend to be reactive and focus on increasing personal resilience or supporting affected individuals, rather than developing proactive, systematic approaches to better prepare learners for clinical practice and to establish a healthier workplace [70, 71]. Exploring how best to support clinicians to thrive in uncertainty offers a potentially valuable contribution to such proactive approaches. For example, organisational approaches might enhance contextual enablers of thriving, such as effective leadership and support from colleagues at work [3]. Education, training, and support might facilitate personal enablers of thriving, such as work engagement and proactivity [2]. Explicitly or implicitly, most existing research considers tolerance of uncertainty to be a relatively stable personal trait [15]. This may be an oversimplification, as individuals can demonstrate different responses to uncertainty in different contexts [72]. Furthermore, a positive relationship between experience and tolerance of uncertainty in doctors has been observed, although the magnitude of the effect was small [73]. A recent review suggested that educational interventions (e.g., problem-based learning, medical humanities) can positively impact tolerance of uncertainty in medical students, although there was marked heterogeneity across studies and inconsistent measurement of tolerance of uncertainty, often using single-item responses or unvalidated measures [74]. A study of individuals with generalised anxiety disorder found that tolerance of uncertainty is amenable to change through behavioural experiments (e.g., by deliberately testing predictions about what will happen in an uncertain situation; [75]). If the same malleability of tolerance of uncertainty exists in individuals without a clinical diagnosis, it is possible that interventions to increase tolerance of uncertainty could facilitate thriving in medical training.

Simply increasing tolerance of uncertainty, however, may be neither achievable nor completely desirable. If it is a stable trait, intervention may have little effect. Further, if tolerance of uncertainty varies across contexts, the impact of any intervention may be context specific. It is also conceivable that too much tolerance of uncertainty is as undesirable as too little. For example, if low tolerance predisposes doctors to over-investigate and overtreat, then excessive tolerance of uncertainty might lead to under-investigation and undertreatment [76]. Indeed, Ilgen et al. [77] ask whether apparent tolerance of uncertainty in clinical practice is an epiphenomenon of ‘competence in context’, that is, tolerance of uncertainty arises as clinicians learn to manage complexity well. They propose that, rather than aiming to develop a generic tolerance of uncertainty, educators should help learners to gauge the uncertainty of the situations they face, tolerating uncertainty when they have the skills to manage it effectively [77]. This is potentially problematic, however, as the current study suggests that greater tolerance of uncertainty increases the likelihood of thriving amongst doctors at the same stage of training. Since this relationship is partially mediated by perceived stress, it may be possible to facilitate thriving by reducing perceived stress in uncertain situations, rather than increasing tolerance of uncertainty. Arousal reappraisal, for example, may facilitate thriving by increasing the likelihood of evaluating the physiological arousal (e.g., elevated heart rate) experienced in uncertain situations as a helpful response that will aid performance (vs. harm it; [78, 79]. Mindset training also has potential to modify core beliefs about stress, such that the stress experienced in uncertainty might be perceived as functional and adaptive rather than debilitating [47, 80].

The current study has several key strengths. Tolerance of uncertainty, wellbeing, and perceived stress were assessed using valid and reliable scales, with cohort scores similar to those reported in relevant population-based validation studies [38, 43, 48]. Participants had graduated from many different medical schools and were working in a range of NHS healthcare settings. The impact of potential confounding variables, such as lifetime stress and ACEs, was accounted for in data analysis. The results enhance our understanding of tolerance of uncertainty, finding that it is not only relevant to mental ill-health, but also to positive wellbeing and perceived performance (i.e., human thriving). Indeed, this study identifies tolerance of uncertainty as a novel enabler of thriving. Furthermore, the mechanisms by which personal and contextual enablers of thriving exert their influence may extend beyond satisfaction of basic psychological needs and challenge appraisals to lowering perceived stress. Previously identified enablers of thriving, such as a proactive personality, supportive co-workers, and effective leadership [2, 3], may also act by reducing perceived stress, potentially through facilitating a subconscious perception of safety [21]. As tolerance of uncertainty and perceived stress only accounted for a proportion of variance in thriving in this study, future research could usefully explore how they interact with these previously identified enablers of thriving.

Despite the aforementioned strengths, caution must be exercised in inferring causation from a cross-sectional study. Although the timescales considered by participants when completing self-report items are consistent with our hypotheses and theoretical framework, it might be considered that lower perceived stress or greater thriving could increase tolerance of uncertainty, or that relationships are bidirectional [52]. Future longitudinal research should therefore explore temporal relationships between tolerance of uncertainty, perceived stress, and thriving. Participants in this study were making the transition to postgraduate training, a time when doctors may experience significant uncertainty [16, 18]. Although the effect of experience on tolerance of uncertainty may be small [73], further research is required to understand whether relationships observed in this study hold true at other stages of a medical career (e.g., after completion of training). The present study explored perceived stress as a potential mediator of the relationship between tolerance of uncertainty and thriving. Further work should test other potential processes in mediating this relationship, such as the role of basic psychological need satisfaction or challenge appraisals [59]. A limitation of self-report questionnaires used in this study is the potential for bias (e.g., social desirability [81]). Where possible, future research should include objective measures. For example, heart rate variability (HRV) parameters demonstrate change under stress [82], *in*tolerance of uncertainty has been associated with reduced high-frequency HRV power during a worry-inducing task [83], and, in a study of clinical reasoning performance in medical students, HRV was associated with self-reported cognitive load and performance under pressure [84]. HRV analysis may thus offer further insight into the relationships between tolerance of uncertainty, perceived stress, and thriving. This study measured self-assessed performance, in keeping with the concept of thriving described in the introduction [1]. However, tolerance of uncertainty and perceived stress may conceivably influence objective performance, or the consistency of self-assessed performance and performance as assessed by others [85]. The addition of objective measures of performance would therefore help explore these relationships in future studies. Finally, thriving may be experienced at a collective level as well as individually [86]. Given the interdependency of multi-professional team members in healthcare services, future research should explore the relationship between tolerance of uncertainty and collective thriving.

## Conclusion

This study investigated whether tolerance of uncertainty enabled human thriving in doctors making the transition from medical school to clinical practice. Furthermore, it examined perceived stress as a potential mediator of this relationship. Results revealed a positive association between tolerance of uncertainty and thriving, and a negative relationship between tolerance of uncertainty and perceived stress. Mediation analysis suggested that the relationship between tolerance of uncertainty and thriving was partially mediated by perceived stress, such that higher tolerance of uncertainty may facilitate thriving, due, at least in part, to lowering perceived stress. This study is the first to identify tolerance of uncertainty as an enabler of human thriving. This has important theoretical implications for thriving research in other domains where uncertainty is commonplace (e.g., sport, military). Within medical education, this study provides empirical support for increasing curricular emphasis on supporting medical students and doctors to develop their capability to tolerate and manage uncertainty. Medical educators should therefore consider how best to prepare doctors in training to thrive in uncertainty. To inform these efforts, future research should explore the mechanisms by which tolerance of uncertainty may influence perceived stress and thriving (e.g., basic psychological needs satisfaction or evoking challenge appraisals), and to examine the utility of interventions aiming to optimise tolerance of uncertainty or decrease perceived stress in uncertain situations.

## Electronic supplementary material

Below is the link to the electronic supplementary material.

Supplementary Material 1

## Data Availability

Anonymised data created during this research are openly available from the University of Bath Research Data Archive at https://doi.org/10.15125/BATH-01433 (Peek, in press).

## Abbreviations

ACEs: Adverse Childhood Experiences
GMC: General Medical Council
GUTS: Generalised Unsafety Theory of Stress
HRV: Heart Rate Variability
NHS: National Health Service
PSS: Perceived Stress Scale
SCSI: Subjective Career Success Inventory
TAMSAD: Tolerance of Ambiguity in Medical Students and Doctors
UK: United Kingdom
WEMWBS: Warwick-Edinburgh Mental Well-Being Scale
WHO: World Health Organisation

## References

[1] Brown DJ, Arnold R, Fletcher D, Standage M. Human thriving: A conceptual debate and literature review. Eur Psychol. 2017;22:167–79.

[2] Kleine AK, Rudolph CW, Zacher H. Thriving at work: A meta-analysis. J Organ Behav. 2019;40(9–10):973–99.

[3] Liu D, Zhang S, Wang Y, Yan Y. The antecedents of thriving at work: A Meta-Analytic review. Front Psychol. 2021;12(August):1–19. 10.3389/fpsyg.2021.659072 PMC8374041 34421715

[4] NHS England. NHS England » NHS health and wellbeing framework [Internet]. 2021 [cited 2025 Feb 27]. Available from: https://www.england.nhs.uk/publication/nhs-health-and-wellbeing-framework/

[5] NHS Survey Coordination Centre. NHS Staff Survey. 2023.

[6] GMC. The state of medical education and practice in the UK. 2023.

[7] Cohidon C, Wild P, Senn N. Job stress among gps: associations with practice organisation in 11 high-income countries. Br J Gen Pract. 2020;70(698):e657–67. 32661010 10.3399/bjgp20X710909 PMC7363272

[8] Shackelton R, Siegrist J, Link C, Marceau L, Von Dem Knesebeck O, Mckinlay J. Work stress of primary care physicians in the US, UK and German health care systems. Soc Sci Med. 2010;71(2):298–304. 20494505 10.1016/j.socscimed.2010.03.043 PMC2885562

[9] Freedman S, Wolf R. The NHS productivity puzzle. Why has hospital activity not increased in line with funding and staffing? 2023.

[10] Gielissen KA, Taylor EP, Vermette D, Doolittle B. Thriving among primary care physicians: a qualitative study. J Gen Intern Med. 2021;36(12):3759–65. 34047922 10.1007/s11606-021-06883-6 PMC8642558

[11] General Medical Council UK. Outcomes for graduates 2018.

[12] Beresford EB. Uncertainty and the shaping of medical decisions. Hastings Cent Rep. 1991;21(4):6–11. 1938352

[13] Farnan JM, Johnson JK, Meltzer DO, Humphrey HJ, Arora VM. Resident uncertainty in clinical decision making and impact on patient care: a qualitative study. Qual Saf Health Care. 2008;17:122–6. 18385406 10.1136/qshc.2007.023184

[14] Hatch S. Uncertainty in medicine. BMJ. 2017;357:j2180. 28495912 10.1136/bmj.j2180

[15] Hillen MA, Gutheil CM, Strout TD, Smets EMA, Han PKJ. Tolerance of uncertainty: conceptual analysis, integrative model, and implications for healthcare. Soc Sci Med. 2017;180:62–75. 28324792 10.1016/j.socscimed.2017.03.024

[16] Cameron A, Millar J, Szmidt N, Hanlon K, Cleland J. Can new Doctors be prepared for practice? A review. Clin Teach. 2014;11(3):188–92. 24802919 10.1111/tct.12127

[17] Lempp H, Cochrane M, Seabrook M, Rees J. Impact of educational preparation on medical students in transition from final year to PRHO year: a qualitative evaluation of final-year training following the introduction of a new Year 5 curriculum in a London medical school. 2004;26(3):276–8. 10.1080/248-0142159042000192046. 10.1080/248-0142159042000192046 15203508

[18] Padley J, Boyd S, Jones A. Walters| lucie. Transitioning from university to postgraduate medical training: A narrative review of work readiness of medical graduates. Heal Sci Rep. 2021;4(2):e270. 10.1002/hsr2.270 PMC8025846 33855193

[19] Schlossberg NK. A model for analyzing human adaptation to transition. Couns Psychol. 1981;9(2).

[20] Carleton RN. Into the unknown: A review and synthesis of contemporary models involving uncertainty. J Anxiety Disord. 2016;39:30–43. 26945765 10.1016/j.janxdis.2016.02.007

[21] Brosschot JF, Verkuil B, Thayer JF. Generalized unsafety theory of stress: unsafe environments and conditions, and the default stress response. Int J Environ Res Public Health. 2018;15(3). 10.3390/ijerph15030464 PMC5877009 29518937

[22] Brosschot JF, Verkuil B, Thayer JF. The default response to uncertainty and the importance of perceived safety in anxiety and stress: an evolution-theoretical perspective. J Anxiety Disord. 2015;41:22–34. 10.1016/j.janxdis.2016.04.012 27259803

[23] Mofrad L, Tiplady A, Payne D, Freeston M. Making friends with uncertainty: experiences of developing a transdiagnostic group intervention targeting intolerance of uncertainty in IAPT. Feasibility, acceptability and implications. Cognitive Behaviour Therapist. Cambridge University; 2020.

[24] Hancock J, Mattick K. Tolerance of ambiguity and psychological well-being in medical training: A systematic review. Med Educ. 2020;54(2):125–37. 31867801 10.1111/medu.14031 PMC7003828

[25] Simons G, Baldwin DS. A critical review of the definition of ‘wellbeing’ for Doctors and their patients in a post Covid-19 era. Int J Soc Psychiatry. 2021;67(8):984–91. 34240644 10.1177/00207640211032259 PMC8592098

[26] World Health Organization. Health promotion glossary of terms 2021. World Health Organ. 2021. 1–44 p.

[27] Thibodeau MA, Carleton RN, Gómez-Pérez L, Asmundson GJG. What if i make a mistake? Intolerance of uncertainty is associated with poor behavioral performance. J Nerv Ment Dis. 2013;201(9):760–6. 23995031 10.1097/NMD.0b013e3182a21298

[28] Carleton RN, Duranceau S, Shulman EP, Zerff M, Gonzales J, Mishra S. Self-reported intolerance of uncertainty and behavioural decisions. J Behav Ther Exp Psychiatry. 2016;51:58–65. 26788617 10.1016/j.jbtep.2015.12.004

[29] Tallentire VR, Smith SE, Skinner J, Cameron HS. Understanding the behaviour of newly qualified Doctors in acute care contexts. Med Educ. 2011;45(10):995–1005. 21916939 10.1111/j.1365-2923.2011.04024.x

[30] Abramowitz JS, Deacon BJ, Whiteside SPH. Exposure therapy for anxiety: Principles and practice, 2nd ed. Exposure therapy for anxiety: Principles and practice, 2nd ed. New York, NY, US: The Guilford Press; 2019. p. xvi, 459–xvi, 459.

[31] Faraji-Rad A, Pham MT. Uncertainty increases the reliance on affect in decisions. J Consum Res. 2017;44(1):1–21.

[32] Morriss J, Tupitsa E, Dodd HF, Hirsch CR. Uncertainty makes me emotional: uncertainty as an elicitor and modulator of emotional States. Front Psychol. 2022;13(March):1–12. 10.3389/fpsyg.2022.777025 PMC8957830 35350739

[33] Simpkin AL, Schwartzstein RM. Tolerating Uncertainty — The next medical revolution?? N Engl J Med. 2016;375(18). 10.1056/NEJMp1606402 27806221

[34] Bachman KH, Freeborn DK. HMO physicians’ use of referrals. Soc Sci Med. 1999;48(4):547–57. 10075179 10.1016/s0277-9536(98)00380-3

[35] Iannello P, Mottini A, Tirelli S, Riva S, Antonietti A. Ambiguity and uncertainty tolerance, need for cognition, and their association with stress. A study among Italian practicing physicians. Med Educ Online. 2017;22(1). 10.1080/10872981.2016.1270009 PMC5328324 28178917

[36] McCarty RJ, Downing ST, Daley ML, Mcnamara JPH, Guastello AD. Relationships between stress appraisals and intolerance of uncertainty with psychological health during early COVID-19 in the USA. 2022. 10.1080/10615806.2022.2075855 35549611

[37] Faul F, Erdfelder E, Lang AG, Buchner A. G*Power 3: A flexible statistical power analysis program for the social, behavioral, and biomedical sciences. Behav Res Methods. 2007;39(2):175–91. 17695343 10.3758/bf03193146

[38] Hancock J, Roberts M, Monrouxe L, Mattick K, Hancock J, Mattick ÁK, et al. Medical student and junior doctors’ tolerance of ambiguity: development of a new scale. Adv Heal Sci Educ. 2015;20:113–30. 10.1007/s10459-014-9510-z 24841480

[39] Strout TD, Hillen M, Gutheil C, Anderson E, Hutchinson R, Ward H, et al. Tolerance of uncertainty: A systematic review of health and healthcare-related outcomes. Patient Educ Couns. 2018;101(9):1518–37. 29655876 10.1016/j.pec.2018.03.030

[40] Tavakol M, Dennick R. Making sense of cronbach’s alpha. Int J Med Educ IJME. 2011;2:53–5. 28029643 10.5116/ijme.4dfb.8dfd PMC4205511

[41] Shockley KM, Ureksoy H, Burcu Rodopman O, Poteat LF, Dullaghan TR. Development of a new scale to measure subjective career success: A mixed-methods study. 2015.

[42] Olckers C, Koekemoer E. Psychometric properties, measurement invariance, and construct validity of the subjective career success inventory. Aust J Psychol. 2022;74(1).

[43] Tennant R, Hiller L, Fishwick R, Platt S, Joseph S, Weich S, et al. The Warwick-Edinburgh mental well-being scale (WEMWBS): development and UK validation. Health Qual Life Outcomes. 2007;5(1):1–13. 18042300 10.1186/1477-7525-5-63 PMC2222612

[44] Brown DJ, Arnold R, Standage M, Fletcher D. A longitudinal examination of thriving in sport performers. Psychol Sport Exerc. 2021;55.

[45] Cohen S, Kamarck T, Mermelstein R. A global measure of perceived stress. J Health Soc Behav. 1983;24(4):385–96. 6668417

[46] Cohen S. Perceived stress in a probability sample of the United States. In: The social psychology of health. Thousand Oaks, CA, US: Sage Publications, Inc; 1988. pp. 31–67. (The Claremont Symposium on Applied Social Psychology.).

[47] Crum AJ, Salovey P, Achor S. Rethinking stress: the role of mindsets in determining the stress response. J Pers Soc Psychol. 2013;104(4):716–33. 23437923 10.1037/a0031201

[48] Cohen S, Janicki-Deverts D. Who’s Stressed? Distributions of Psychological Stress in the United States in Probability Samples. 2012.

[49] Hughes K, Lowey H, Quigg Z, Bellis MA. Relationships between adverse childhood experiences and adult mental well-being: results from an english National household survey. BMC Public Health. 2016;16(1):1–11. 26940088 10.1186/s12889-016-2906-3 PMC4778324

[50] Wade R, Becker BD, Bevans KB, Ford DC, Forrest CB. Development and evaluation of a short adverse childhood experiences measure. Am J Prev Med. 2017;52(2):163–72. 27865652 10.1016/j.amepre.2016.09.033 PMC5596508

[51] Kim H-Y. Statistical notes for clinical researchers: assessing normal distribution (2) using skewness and kurtosis. Restor Dent Endod. 2013;38(1):52. 23495371 10.5395/rde.2013.38.1.52 PMC3591587

[52] Tabachnick BG, Fidell LS. Using multivariate statistics. 7th editio. Upper Saddle River: Pearson; 2019.

[53] Hayes A. Introduction to mediation, moderation, and conditional process analysis: A regression-based approach. 3rd Editio. New York: The Guilford Press; 2022.

[54] Keister ACC. Thriving teams and change agility: leveraging a collective state to create organization agility. Res Organ Chang Dev. 2014;22:299–333.

[55] Walumbwa FO, Muchiri MK, Misati E, Wu C, Meiliani M. Inspired to perform: A multilevel investigation of antecedents and consequences of thriving at work. J Organ Behav. 2018;39(3):249–61.

[56] Health Education England. NHS Staff and Learners ’ Mental Wellbeing Commission. 2019;(February):1–96.

[57] Han PKJ, Klein WMP, Arora NK. Varieties of uncertainty in health care: A conceptual taxonomy. Med Decis Mak. 2011;31(6):828–38. 10.1177/0272989X11393976 PMC3146626 22067431

[58] Yardley S, Westerman M, Bartlett M, Mark Walton J, Smith J, Peile E, et al. The do’s, don’t and don’t knows of supporting transition to more independent practice. Perspect Med Educ. 2018;7:8–22. 10.1007/s40037-018-0403-3 PMC5807269 29383578

[59] Brown DJ, Arnold R, Standage M, Turner JE, Fletcher D. The prediction of thriving in elite sport: A prospective examination of the role of psychological need satisfaction, challenge appraisal, and salivary biomarkers. J Sci Med Sport. 2021;24(4):373–9. 33077401 10.1016/j.jsams.2020.09.019

[60] Shihata S, McEvoy PM, Mullan BA, Carleton RN. Intolerance of uncertainty in emotional disorders: what uncertainties remain? J Anxiety Disord. 2016;41:115–24. 27212227 10.1016/j.janxdis.2016.05.001

[61] Wilson EJ, Abbott MJ, Norton AR. The impact of psychological treatment on intolerance of uncertainty in generalized anxiety disorder: A systematic review and meta-analysis. J Anxiety Disord. 2023;97(May):102729. 37271039 10.1016/j.janxdis.2023.102729

[62] Buhr K, Dugas MJ. The intolerance of uncertainty scale: psychometric properties of the english version. Behav Res Ther. 2002;40(8):931–45. 12186356 10.1016/s0005-7967(01)00092-4

[63] Einstein DA. Extension of the transdiagnostic model to focus on intolerance of uncertainty: A review of the literature and implications for treatment. Clin Psychol Sci Pract. 2014;21(3):280. 10.1111/cpsp.12077 PMC4204511 25400336

[64] Morriss J, Zuj DV, Mertens G. The role of intolerance of uncertainty in classical threat conditioning: recent developments and directions for future research. Int J Psychophysiol. 2021;166:116–26. 34097936 10.1016/j.ijpsycho.2021.05.011

[65] Seery MD. The biopsychosocial model of challenge and threat: using the heart to measure the Mind. Soc Personal Psychol Compass. 2013;7(9):637–53.

[66] Hase A, O’Brien J, Moore LJ, Freeman P. The relationship between challenge and threat States and performance: A systematic review. Sport Exerc Perform Psychol. 2019;8(2):123–44.

[67] McLoughlin E, Arnold R, Moore LJ. The tendency to appraise stressful situations as more of a threat is associated with poorer health and well-being. Stress Heal. 2024;40(3):1–7. 10.1002/smi.3358 38108652

[68] Bajorek Z, Holmes J. Health and Wellbeing Interventions in Healthcare A rapid evidence review. 2020.

[69] Cohen C, Pignata S, Bezak E, Tie M, Childs J. Workplace interventions to improve well-being and reduce burnout for nurses, physicians and allied healthcare professionals: a systematic review. BMJ Open. 2023;13(6):1–23. 10.1136/bmjopen-2022-071203 PMC10314589 37385740

[70] Montgomery A, Panagopoulou E, Esmail A, Richards T, Maslach C. Burnout in healthcare: the case for organisational change. BMJ. 2019. 10.1136/bmj.l4774 31362957

[71] West M. Creating a Workplace where NHS Staff can Flourish [Internet]. The Kings Fund. 2016 [cited 2019 Dec 28]. Available from: https://www.kingsfund.org.uk/blog/2016/01/creating-workplace-where-staff-can-flourish

[72] Durrheim K, Foster D. Tolerance of ambiguity as a content specific construct. Pers Individ Dif. 1997;22(5):741–50.

[73] Begin AS, Hidrue M, Lehrhoff S, del Carmen MG, Armstrong K, Wasfy JH. Factors associated with physician tolerance of uncertainty: an observational study. J Gen Intern Med. 2021;1. 10.1007/s11606-021-06776-8 PMC8074695 33904030

[74] Patel P, Hancock J, Rogers M, Pollard SR. Improving uncertainty tolerance in medical students: A scoping review. 2022. 10.1111/medu.14873 PMC9796811 35797009

[75] Dugas MJ, Sexton KA, Hebert EA, Shafran R. Behavioral experiments for intolerance of uncertainty: A randomized clinical trial for adults with generalized anxiety disorder. Behav Ther. 2022;53(6):1147–60. 36229113 10.1016/j.beth.2022.05.003

[76] Reis-Dennis S, Gerrity MS, Geller G. Tolerance for uncertainty and professional development: a normative analysis. J Gen Intern Med. 2021;36(8):2408–13. 33532966 10.1007/s11606-020-06538-y PMC7853704

[77] Ilgen JS, Watsjold BK, Regehr G. Is uncertainty tolerance an epiphenomenon? Med Educ. 2022;56(12):1150–2. 36124815 10.1111/medu.14938

[78] Moore LJ, Samuel J, Wilson, Mark R, Freeman. Paul. Reappraising threat: how to optimize performance under pressure. J Sport Exerc Psychol. 2015;(3):339–43. 10.1123/jsep.2014-0186 26265345

[79] Sammy N, Anstiss PA, Moore LJ, Freeman P, Wilson MR, Vine SJ. The effects of arousal reappraisal on stress responses, performance and attention. Anxiety Stress Coping. 2017;30(6):619–29. 28535726 10.1080/10615806.2017.1330952

[80] Jamieson JP, Crum AJ, Goyer JP, Marotta ME, Akinola M. Optimizing stress responses with reappraisal and mindset interventions: an integrated model. Anxiety Stress Coping. 2018;31(3):245–61. 29471669 10.1080/10615806.2018.1442615

[81] Latkin CA, Edwards C, Davey-Rothwell MA, Tobin KE, Latkin CA. The relationship between social desirability bias and self-reports of health, substance use, and social network factors among urban substance users in baltimore, Maryland HHS public access. Addict Behav. 2017;73:133–6. 28511097 10.1016/j.addbeh.2017.05.005 PMC5519338

[82] Kim HG, Cheon EJ, Bai DS, Lee YH, Koo BH. Stress and heart rate variability: A meta-analysis and review of the literature. Psychiatry Investig. 2018;15(3):235–45. 10.30773/pi.2017.08.17 PMC5900369 29486547

[83] Deschênes SS, Dugas MJ, Gouin JP. Intolerance of uncertainty, worry catastrophizing, and heart rate variability during worry-inducing tasks. Pers Individ Dif. 2016;90:199–204.

[84] Solhjoo S, Haigney MC, McBee E, van Merrienboer JJG, Schuwirth L, Artino AR, et al. Heart rate and heart rate variability correlate with clinical reasoning performance and Self-Reported measures of cognitive load. Sci Rep 2019 91. 2019;9(1):1–9. 10.1038/s41598-019-50280-3 PMC6789096 31604964

[85] Andrade HL. A critical review of research on student Self-Assessment. Front Educ. 2019;4(August):1–13.

[86] McGuire CS, Brown DJ, McEwan D, Arnold R, Martin LJ. Thriving together: conceptual and methodological considerations for examining thriving in interdependent sport. Int Rev Sport Exerc Psychol. 2023;1–24.

