# Supplementary material for: Greater tolerance of uncertainty facilitates thriving in doctors entering postgraduate training
Source: BMC Med Educ. 2025 Jul 16;25:1062. doi: 10.1186/s12909-025-07645-2 (PMC12266227; doi:10.1186/s12909-025-07645-2)
Supplement: Supplementary file 1 — Supplementary Material 1 [file 12909_2025_7645_MOESM1_ESM.docx]

**Study questionnaire**

| Your age (Years) | | | | |  | | | | |
| --- | --- | --- | --- | --- | --- | --- | --- | --- | --- |
| Medical school awarding primary qualification | | | | |  | | | | |
| Year of qualification | | | | |  | | | | |
| Your sex | | | | | Male  Female  Prefer to self-describe  Prefer not to say | | | ⭘  ⭘  ⭘ ___________  ⭘ | |
| Do you consider yourself to have a disability? | | | | | Yes  No  Prefer not to say | | | ⭘  ⭘  ⭘ | |
| Do you have any long-term medical conditions? | | | | | Yes  No | | | ⭘  ⭘ | |
|  | | | | | | | | | |
| The next two questions are about potentially adverse experiences in childhood:   - When growing up, did you live with anyone who was a problem drinker or alcoholic? - Did parents or adults in your home swear at you, insult you, or put you down more than once? | | | | | Yes  No  Yes  No | | | ⭘  ⭘  ⭘  ⭘ | |
| The questions below ask about the stress you have experienced throughout your life so far and the stress you are currently experiencing. There are no right or wrong answers, please answer honestly! | | | | | | | | | |
| How much stress have you experienced *throughout your life?* | | | | | | | | | |
| None  0 | 1 | 2 | 3 | | | 4 | 5 | | Extreme amount  6 |
| ⭘ | ⭘ | ⭘ | ⭘ | | | ⭘ | ⭘ | | ⭘ |
| How much stress are you experiencing in your life *right now?* | | | | | | | | | |
| None  0 | 1 | 2 | 3 | | | 4 | 5 | | Extreme amount  6 |
| ⭘ | ⭘ | ⭘ | ⭘ | | | ⭘ | ⭘ | | ⭘ |
| What is the primary source of stress in your life *right now?* | | | |  | | | | | |
| How stressful do you perceive this to be*?* | | | | | | | | | |
| Not stressful  0 | 1 | 2 | 3 | | | 4 | 5 | | Extremely stressful  6 |
| ⭘ | ⭘ | ⭘ | ⭘ | | | ⭘ | ⭘ | | ⭘ |

**Tolerance of Ambiguity in Medical Students and Doctors (TAMSAD)
Hancock et al. (2015)**

Below are some statements about medical practice. Please tick the box that best describes your point of view.

| **Statement** | **strongly disagree** | **disagree** | **neutral** | **agree** | **strongly agree** |
| --- | --- | --- | --- | --- | --- |
| I would enjoy tailoring treatments to individual patient problems | ⭘ | ⭘ | ⭘ | ⭘ | ⭘ |
| I have a lot of respect for consultants who always come up with a definite answer | ⭘ | ⭘ | ⭘ | ⭘ | ⭘ |
| I would be comfortable if a clinical teacher set me a vague assignment or task | ⭘ | ⭘ | ⭘ | ⭘ | ⭘ |
| A good clinical teacher is one who challenges your way of looking at clinical problems | ⭘ | ⭘ | ⭘ | ⭘ | ⭘ |
| What we are used to is always preferable to what is unfamiliar | ⭘ | ⭘ | ⭘ | ⭘ | ⭘ |
| I feel uncomfortable when people claim that something is ‘absolutely certain’ in medicine | ⭘ | ⭘ | ⭘ | ⭘ | ⭘ |
| A doctor who leads an even, regular work life with few surprises, really has a lot to be grateful for | ⭘ | ⭘ | ⭘ | ⭘ | ⭘ |
| I think in medicine it is important to know exactly what you are talking about at all times | ⭘ | ⭘ | ⭘ | ⭘ | ⭘ |
| I feel comfortable that in medicine there is often no right or wrong answer | ⭘ | ⭘ | ⭘ | ⭘ | ⭘ |
| A patient with multiple diseases would make a doctor’s job more interesting | ⭘ | ⭘ | ⭘ | ⭘ | ⭘ |
| I am uncomfortable that a lack of medical knowledge about some diseases means we can’t help some patients | ⭘ | ⭘ | ⭘ | ⭘ | ⭘ |
| The unpredictability of a patient’s response to medication would bring welcome complexity to a doctor’s role | ⭘ | ⭘ | ⭘ | ⭘ | ⭘ |
| It is important to appear knowledgeable to patients at all times | ⭘ | ⭘ | ⭘ | ⭘ | ⭘ |
| Being confronted with contradictory evidence in clinical practice makes me feel uncomfortable | ⭘ | ⭘ | ⭘ | ⭘ | ⭘ |
| I like the mystery that there are somethings in medicine we’ll never know | ⭘ | ⭘ | ⭘ | ⭘ | ⭘ |
| Variation between individual patients is a frustrating aspect of medicine | ⭘ | ⭘ | ⭘ | ⭘ | ⭘ |
| I find it frustrating when I can’t find the answer to a clinical question | ⭘ | ⭘ | ⭘ | ⭘ | ⭘ |
| I am apprehensive when faced with a new clinical situation or problem | ⭘ | ⭘ | ⭘ | ⭘ | ⭘ |
| I feel uncomfortable knowing that many of our most important clinical decisions are based upon insufficient information | ⭘ | ⭘ | ⭘ | ⭘ | ⭘ |
| No matter how complicated the situation, a good doctor will be able to arrive at a yes or no answer | ⭘ | ⭘ | ⭘ | ⭘ | ⭘ |
| I feel uncomfortable when textbooks or experts are factually incorrect | ⭘ | ⭘ | ⭘ | ⭘ | ⭘ |
| There is really no such thing as a clinical problem that can’t be solved | ⭘ | ⭘ | ⭘ | ⭘ | ⭘ |
| I like the challenge of being thrown in the deep end with different medical situations | ⭘ | ⭘ | ⭘ | ⭘ | ⭘ |
| It is more interesting to tackle a complicated clinical problem that to solve a simple one | ⭘ | ⭘ | ⭘ | ⭘ | ⭘ |
| I enjoy the process of working with a complex clinical problem and making it more manageable | ⭘ | ⭘ | ⭘ | ⭘ | ⭘ |
| A good job is one where what is to be done and how it is to be done are always clear | ⭘ | ⭘ | ⭘ | ⭘ | ⭘ |
| To me, medicine is black and white | ⭘ | ⭘ | ⭘ | ⭘ | ⭘ |
| The beauty of medicine is that it’s always evolving and changing | ⭘ | ⭘ | ⭘ | ⭘ | ⭘ |
| I would be comfortable to acknowledge the limits of my medical knowledge to patients | ⭘ | ⭘ | ⭘ | ⭘ | ⭘ |

**Perceived Stress Scale (PSS)
Cohen et al. (1983)**

The questions in this scale ask you about your feelings and thoughts during **the last month**. In each case, please indicate how often you felt or thought a certain way.

| **In the last month, how often** | **never** | **almost never** | **sometimes** | | **fairly often** | **very often** |
| --- | --- | --- | --- | --- | --- | --- |
| have you been upset because of something that happened unexpectedly? | ⭘ | ⭘ | ⭘ | ⭘ | | ⭘ |
| have you felt that you were unable to control the important things in your life? | ⭘ | ⭘ | ⭘ | ⭘ | | ⭘ |
| have you felt nervous and "stressed"? | ⭘ | ⭘ | ⭘ | ⭘ | | ⭘ |
| have you felt confident about your ability to handle your personal problems? | ⭘ | ⭘ | ⭘ | ⭘ | | ⭘ |
| have you felt that things were going your way? | ⭘ | ⭘ | ⭘ | ⭘ | | ⭘ |
| have you found that you could not cope with all the things that you had to do? | ⭘ | ⭘ | ⭘ | ⭘ | | ⭘ |
| have you been able to control irritations in your life? | ⭘ | ⭘ | ⭘ | ⭘ | | ⭘ |
| have you felt that you were on top of things? | ⭘ | ⭘ | ⭘ | ⭘ | | ⭘ |
| have you been angered because of things that were outside of your control? | ⭘ | ⭘ | ⭘ | ⭘ | | ⭘ |
| how often have you felt difficulties were piling up so high that you could not overcome them? | ⭘ | ⭘ | ⭘ | ⭘ | | ⭘ |

**Warwick-Edinburgh Mental Well-Being Scale (WEMWBS)
Tennant et al. (2007)**

Below are some statements about feelings and thoughts. Please tick the box that best describes your experience of each over the last 2 weeks.

| **Statements** | **None of the time** | **Rarely** | **Some of the time** | **Often** | **All of the time** |
| --- | --- | --- | --- | --- | --- |
| I’ve been feeling optimistic about the future | ⭘ | ⭘ | ⭘ | ⭘ | ⭘ |
| I’ve been feeling useful | ⭘ | ⭘ | ⭘ | ⭘ | ⭘ |
| I’ve been feeling relaxed | ⭘ | ⭘ | ⭘ | ⭘ | ⭘ |
| I’ve been feeling interested in other people | ⭘ | ⭘ | ⭘ | ⭘ | ⭘ |
| I’ve had energy to spare | ⭘ | ⭘ | ⭘ | ⭘ | ⭘ |
| I’ve been dealing with problems well | ⭘ | ⭘ | ⭘ | ⭘ | ⭘ |
| I’ve been thinking clearly | ⭘ | ⭘ | ⭘ | ⭘ | ⭘ |
| I’ve been feeling good about myself | ⭘ | ⭘ | ⭘ | ⭘ | ⭘ |
| I’ve been feeling close to other people | ⭘ | ⭘ | ⭘ | ⭘ | ⭘ |
| I’ve been feeling confident | ⭘ | ⭘ | ⭘ | ⭘ | ⭘ |
| I’ve been able to make up my own mind about things | ⭘ | ⭘ | ⭘ | ⭘ | ⭘ |
| I’ve been feeling loved | ⭘ | ⭘ | ⭘ | ⭘ | ⭘ |
| I’ve been interested in new things | ⭘ | ⭘ | ⭘ | ⭘ | ⭘ |
| I’ve been feeling cheerful | ⭘ | ⭘ | ⭘ | ⭘ | ⭘ |

**Subjective Career Success Inventory (SCSI)
Shockley et al. (2015)**

| **Considering my career as a whole…** | **strongly disagree** | **disagree** | **neutral** | | **agree** | **strongly agree** |
| --- | --- | --- | --- | --- | --- | --- |
| …my supervisors have told me I do a good job. | ⭘ | ⭘ | ⭘ | ⭘ | | ⭘ |
| …the organisations I worked for have recognised me as a good performer. | ⭘ | ⭘ | ⭘ | ⭘ | | ⭘ |
| …I have been recognised for my contributions. | ⭘ | ⭘ | ⭘ | ⭘ | | ⭘ |
| …I am proud of the quality of the work I have produced. | ⭘ | ⭘ | ⭘ | ⭘ | | ⭘ |
| …I have met the highest standards of quality in my work. | ⭘ | ⭘ | ⭘ | ⭘ | | ⭘ |
| …I have been known for the high quality of my work. | ⭘ | ⭘ | ⭘ | ⭘ | | ⭘ |
| …I think my work has been meaningful. | ⭘ | ⭘ | ⭘ | ⭘ | | ⭘ |
| …I believe my work has made a difference. | ⭘ | ⭘ | ⭘ | ⭘ | | ⭘ |
| …the work I have done has contributed to society. | ⭘ | ⭘ | ⭘ | ⭘ | | ⭘ |
| …decisions that I have made have impacted my organisation. | ⭘ | ⭘ | ⭘ | ⭘ | | ⭘ |
| …the organisations I have worked for have considered my opinion regarding important issues. | ⭘ | ⭘ | ⭘ | ⭘ | | ⭘ |
| …others have taken my advice into account when making important decisions. | ⭘ | ⭘ | ⭘ | ⭘ | | ⭘ |
| …I have been able to pursue work that meets my personal needs and preferences. | ⭘ | ⭘ | ⭘ | ⭘ | | ⭘ |
| …I have felt as though I am in charge of my own career. | ⭘ | ⭘ | ⭘ | ⭘ | | ⭘ |
| …I have chosen my own career path | ⭘ | ⭘ | ⭘ | ⭘ | | ⭘ |
| …I have been able to spend the amount of time I want with my friends and family. | ⭘ | ⭘ | ⭘ | ⭘ | | ⭘ |
| …I have been able to have a satisfying life outside of work. | ⭘ | ⭘ | ⭘ | ⭘ | | ⭘ |
| …I have been able to be a good employee while maintaining quality non-work relationships. | ⭘ | ⭘ | ⭘ | ⭘ | | ⭘ |
| …I have expanded my skill sets to perform better. | ⭘ | ⭘ | ⭘ | ⭘ | | ⭘ |
| …I have stayed current with changes in my field | ⭘ | ⭘ | ⭘ | ⭘ | | ⭘ |
| …I have continuously improved by developing my skill set. | ⭘ | ⭘ | ⭘ | ⭘ | | ⭘ |
| …my career is personally satisfying. | ⭘ | ⭘ | ⭘ | ⭘ | | ⭘ |
| …I am enthusiastic about my career. | ⭘ | ⭘ | ⭘ | ⭘ | | ⭘ |
| …I have found my career quite interesting. | ⭘ | ⭘ | ⭘ | ⭘ | | ⭘ |
